# Enhancing generalized anxiety disorder diagnosis precision: MSTCNN model utilizing high-frequency EEG signals

**DOI:** 10.3389/fpsyt.2023.1310323

**Published:** 2023-12-21

**Authors:** Wei Liu, Gang Li, Ziyi Huang, Weixiong Jiang, Xiaodong Luo, Xingjuan Xu

**Affiliations:** ^1^College of Computer Science and Technology, Zhejiang Normal University, Jinhua, China; ^2^College of Mathematical Medicine, Zhejiang Normal University, Jinhua, China; ^3^School of Advanced Technology, Xi'an Jiaotong-Liverpool University, Suzhou, China; ^4^The Second Hospital of Jinhua, Jinhua, China

**Keywords:** generalized anxiety disorder (GAD), electroencephalogram (EEG), convolutional neural network (CNN), attention mechanisms, deep learning

## Abstract

Generalized Anxiety Disorder (GAD) is a prevalent mental disorder on the rise in modern society. It is crucial to achieve precise diagnosis of GAD for improving the treatments and averting exacerbation. Although a growing number of researchers beginning to explore the deep learning algorithms for detecting mental disorders, there is a dearth of reports concerning precise GAD diagnosis. This study proposes a multi-scale spatial–temporal local sequential and global parallel convolutional model, named MSTCNN, which designed to achieve highly accurate GAD diagnosis using high-frequency electroencephalogram (EEG) signals. To this end, 10-min resting EEG data were collected from 45 GAD patients and 36 healthy controls (HC). Various frequency bands were extracted from the EEG data as the inputs of the MSTCNN. The results demonstrate that the proposed MSTCNN, combined with the attention mechanism of Squeeze-and-Excitation Networks, achieves outstanding classification performance for GAD detection, with an accuracy of 99.48% within the 4–30 Hz EEG data, which is competitively related to state-of-art methods in terms of GAD classification. Furthermore, our research unveils an intriguing revelation regarding the pivotal role of high-frequency band in GAD diagnosis. As the frequency band increases, diagnostic accuracy improves. Notably, high-frequency EEG data ranging from 10–30 Hz exhibited an accuracy rate of 99.47%, paralleling the performance of the broader 4–30 Hz band. In summary, these findings move a step forward towards the practical application of automatic diagnosis of GAD and provide basic theory and technical support for the development of future clinical diagnosis system.

## 1 Introduction

Generalized Anxiety Disorder (GAD) is a common psychiatric disorder characterized by persistent anxiety, irritability, sleep disturbances, and nervousness (1). In addition, patients with GAD often have physical symptoms such as palpitations, dry mouth, and excessive sweating (2). Recently, the incidence of GAD has significantly increased and has become a global health issue. It is reported that the global rate of the people with anxiety disorder was 26% in 2020, and the growth rate has accelerated compared to previous years (3). The lifetime prevalence rate of GAD in the general population is as high as 5% (4). Females have a much higher probability of developing this disorder compared to males (5). GAD not only brings negative impacts on the psychological and physical health of patients but also has the potential to seriously affect their daily functioning, social interaction, and quality of life.

The etiological factor of GAD is exceedingly intricate, encompassing the interplay of genetic, biological, and psychosocial factors (6, 7). The complex etiologies of GAD emphasize the need for a targeted treatment approach. Therefore, timeous diagnosis combined with effective treatment is crucial to avoid GAD becoming more severe and harder to treat (8). Currently, clinical diagnosis of GAD mainly relies on clinical assessment and subjective scales (9). These methods are highly subjective and rely heavily on accurate diagnosis by the psychiatrists and accurate self-reporting by the patients, which may easily lead to inconsistency and inaccuracy in diagnosis and assessing efficacy. Therefore, it is crucial to seek objective and precise diagnostic methods for GAD.

With the continuous developments of psychiatric neuroscience, a range of neuroimaging techniques have been applied to the study of psychiatric diseases including electroencephalogram (EEG) (10, 11), magnetoencephalography (MEG) (12), near-infrared spectroscopy (NIRS) (13), and functional magnetic resonance imaging (fMRI) (14). Among these techniques, EEG has excellent timing resolution and high time sensitivity, while being non-invasive and simple to operate (15, 16). EEG can record and measure the brain activity, offering valuable insights into its dynamic functioning (17). In recent years, the application of EEG to GAD has been continuously explored to help uncover the complex neuro-electrophysiological mechanism and provide more effective detection methods. Previous studies have utilized EEG to observe changes in the brain of GAD patients, such as increased brain activity (18) and alterations in brain network structure (19). Furthermore, by extracting various types of EEG features, such as functional connectivity (19), power spectral density (20), and correlation dimension (21), researchers found significant differences in features between GAD patients and healthy controls. Until now, EEG has been widely used to assist in the diagnosis of various psychiatric disorders, such as anxiety (22, 23), depression (24, 25), obsessive-compulsive disorder (26, 27), Alzheimer’s (28, 29), schizophrenia (30, 31). These studies imply that EEG is a valuable and promising neuroimaging technique in the diagnosis of GAD.

Prior research related to mental disorder detection that combines artificial intelligence and EEG can be mainly divided into two categories. On the one hand, some researchers extract diverse EEG features (32–34), utilizing machine learning models for classification. This strategy strongly relies on the classification performances of the extracted features and the adaptability of the machine learning models. On the other hand, existence of researchers building deep learning models and using EEG signals as the inputs for classification. Deep learning can overcome the shortcomings of high feature dependence and limited shallow models. It streamlines processing by enabling automated end-to-end learning, integrating feature extraction and classification. Deep learning has demonstrated significant success in the processing of complex data (35). Due to the excellent end-to-end learning and ability to effectively utilize data hierarchies, convolutional neural network (CNN) has emerged as a widely favored architecture in deep learning-EEG research (36). For instance, Abdulhakim employed three different deep learning models: CNN, long short term memory (LSTM), CNN + LSTM, and achieved the highest accuracy of 92.86% for social anxiety disorder identification with CNN + LSTM model (37). Although the combination of EEG and deep learning has shown remarkable success in variety of fields (38–40), according to our previous survey, it is rarely utilized in GAD diagnosis, which highlights the urgent need for enhanced diagnostic methods in this specific domain.

Given the challenging low signal-to-noise ratio of EEG signals and complex spatiotemporal dynamic patterns, the importance of feature extraction in deep learning is magnified. As an efficient and rapid EEG signal feature extraction tool, CNN plays a powerful role in the field of EEG signal analysis. For EEG signals, traditional time-frequency domain feature extraction methods encounter challenges to fully capture the intricate details. Consequently, adopting the spatial–temporal joint feature extraction method has a stronger signal representation ability in CNN model (41). Moreover, multi-scale convolution of CNN has been emphasized in EEG feature extraction. This technique can capture different levels of features at different scales, thereby enhancing the characterization ability of the model. Researchers have successfully applied multi-scale convolution to feature extraction, yielding favorable outcomes (42–44). For instance, Wu et al. introduced a parallel multi-scale filter bank CNN for EEG classification, and achieved excellent classification performance (44). To further elevate CNN performance, multi-scale convolution was introduced into the spatial–temporal feature extraction for GAD diagnosis.

In this study, we propose an end-to-end deep learning model architecture called MSTCNN based on multi-scale spatial–temporal convolution to facilitate in the precise diagnosis of GAD. To ensure the effectiveness of MSTCNN, we conducted a sequence of ablation experiments to validate the efficacy of our selection strategy in model design. In addition, we try to use MSTCNN to reveal the key frequency bands of GAD, which helps us understand the potential differences of GAD in different frequency bands of EEG signals. Our research strives to present a viable approach for the precise diagnosis of GAD.

## 2 Materials and methods

### 2.1 Subjects

A total of 45 patients with GAD (13 males, 32 females, age: 22–55 years, 41.8 ± 9.4 years) and 36 healthy controls (HC) (11 males, 25 females, age: 21–57 years, 36.9 ± 11.3 years) were enrolled in this study, and there was no statistically significant difference in age between GAD and HC. All patients were diagnosed by the specialized psychiatrists and meet the DSM-5-TR criteria for GAD diagnosis. And all subjects should complete the questionnaire of Hamilton Anxiety Rating Scale (HAMA) and meet the following criteria: HAMA scores ≥14 for GAD; HAMA scores ≤7 for HC. Additionally, GAD patients had no other comorbidities (such as depression and other disorders). The average HAMA score in the GAD group was 27.1 ± 9.0, and in the HC group was 2.3 ± 0.9. Moreover, each participant was required to meet stringent EEG data collection requirements: (1) no other psychiatric disorders and brain damage; (2) right-handed; (3) no drug and alcohol abuse; (4) not stay up late the day before the EEG data collection; (5) no smoking, coffee and strong tea before eight hours of EEG data collection. The entire experiment received approval from the Ethics Committee of Zhejiang Normal University, and all participants provided a written informed consent form before the experiment.

### 2.2 EEG data collection and preprocessing

Participants were asked to close eyes, stay awake and stationary, and reduce head and body movements and eye movements to reduce interference from ocular and electromyography. Every participant would record clinical resting EEG for 10 min. The EEG acquisition device is Nicolet EEG TS215605. Following the international 10–20 system, 16 electrodes were chosen, namely Fp1, Fp2, F3, F4, C3, C4, P3, P4, O1, O2, F7, F8, T7, T8, P7, and P8. The reference electrode refers to the left and right mastoid electrodes. The sampling frequency is 250 Hz, and the impedance of each electrode is controlled below 5kΩ. The whole experiment took place within the professional EEG laboratory of the local hospital.

Then, the EEGLAB embedded in MATLAB R2021a was used to preprocess EEG. Firstly, the original EEG signal was down-sampled from 250 Hz to 125 Hz, and the signal was filtered by 4–30 Hz bandpass using a 4-order Butterworth filter. Secondly, fast independent component analysis (ICA) was used to remove EEG artifacts. Then, 4 s of continuous EEG signals were extracted as an EEG sample, resulting in a total of 5,371 samples for GAD and 4,018 samples for HC. Finally, the same bandpass filter was used to divide the EEG signal into five basic bands: Theta (4–8 Hz), Alpha1 (8–10 Hz), Alpha2 (10–13 Hz), Beta1 (13–20 Hz), Beta2 (20–30 Hz), and three extended bands: 13-30 Hz, 10-30 Hz, 8-30 Hz.

### 2.3 MSTCNN model

**Figure 1:**
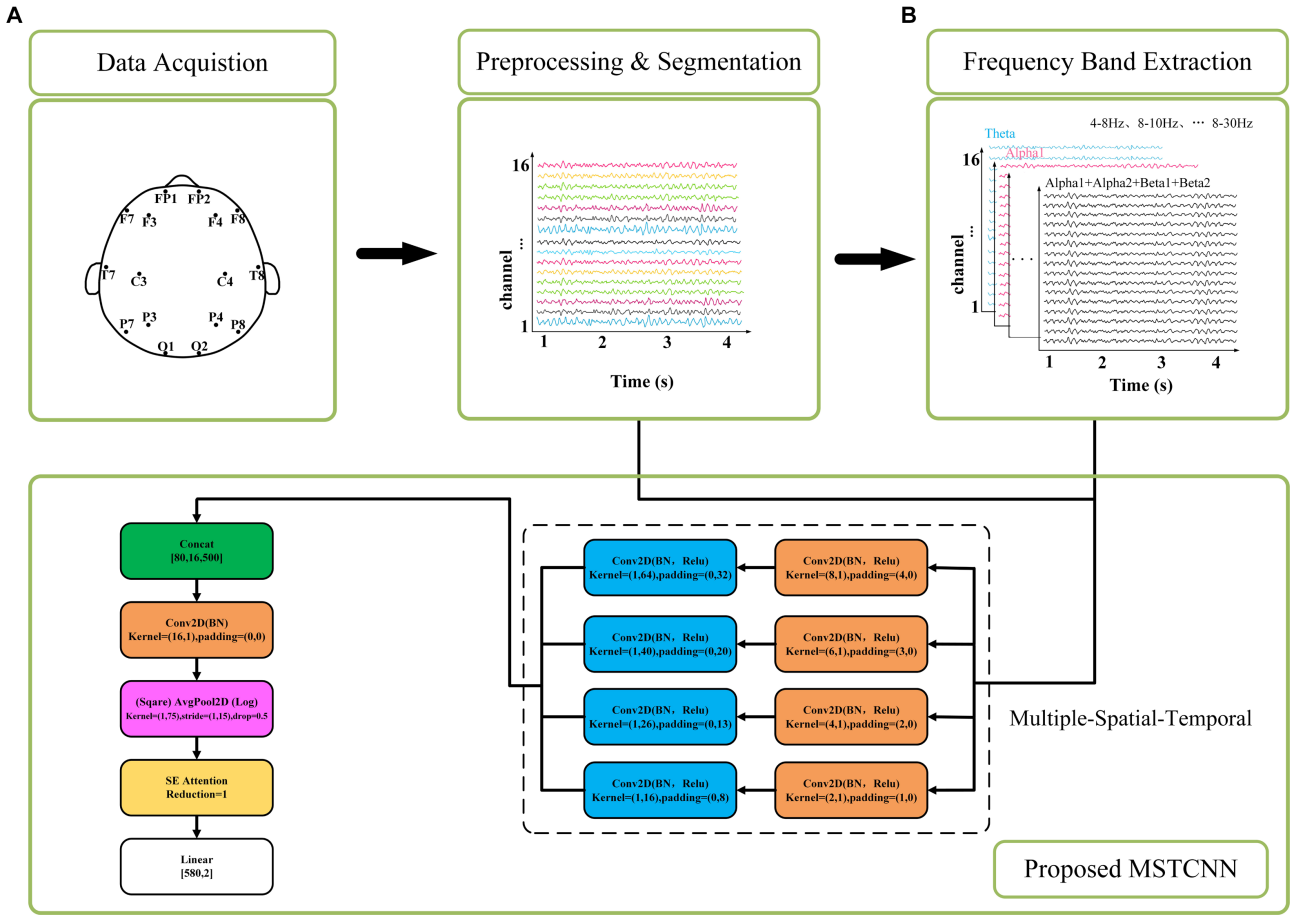
The corresponding network architecture of the MSTCNN. **(A)** represents the input of raw EEG signal at 4–30 Hz. **(B)** represents the input of different frequency bands for comparison.

In this study, we proposed an innovative deep learning model named MSTCNN for GAD detection, which incorporates multi-scale spatial–temporal local sequential and global parallel convolutions. This architecture is further enhanced through the integration of an attention mechanism strategy. Its basic flow is shown in Figure 1. Detailed parameters of MSTCNN can be found in Table 1. The framework of MSTCNN can be divided into a feature extraction layer and a feature classification layer. (1) The feature extraction layer aims to learn and extract the most representative features from the original EEG signal, capturing the crucial information of the input data to provide support for subsequent classification tasks. This layer includes spatiotemporal feature extraction, spatial feature extraction, and attention mechanism modules. The spatiotemporal feature extraction layer adopts multi-scale convolution, which helps to effectively extract spatiotemporal features at different scales. The spatial feature extraction layer is used to extract spatial features and reduce the dimensionality of the data. Prior to being fed into the fully connected layer, attention mechanism modules are added to enhance attention to important features and further improve model performance. (2) The feature classification layer primarily consists of nonlinear function, Dropout and pooling layer, which is used to enhance the nonlinear expressive ability, mitigate overfitting risks, and reduce data dimensionality.

**Table 1:** Parameters of proposed MSTCNN architecture.

Layer	Filters	Size	Stride	Output	Padding
Input				(16, 500)	
Reshape				(1, 16, 500)	
SpaConv1	10	(8, 1)	(1, 1)	(10, 16, 500)	Same
BatchNorm (ReLU)				(10, 16, 500)	
TemConv1	20	(1, 64)	(1, 1)	(20, 16, 500)	Same
BatchNorm (ReLU)				(20, 16, 500)	
SpaConv2	10	(6, 1)	(1, 1)	(10, 16, 500)	Same
BatchNorm (ReLU)				(10, 16, 500)	
TemConv2	20	(1, 40)	(1, 1)	(20, 16, 500)	Same
BatchNorm (ReLU)				(20, 16, 500)	
SpaConv3	10	(4, 1)	(1, 1)	(10, 16, 500)	Same
BatchNorm (ReLU)				(10, 16, 500)	
TemConv3	20	(1, 26)	(1, 1)	(20, 16, 500)	Same
BatchNorm (ReLU)				(20, 16, 500)	
SpaConv4	10	(2, 1)	(1, 1)	(10, 16, 500)	Same
BatchNorm (ReLU)				(10, 16, 500)	
TemConv4	20	(1, 16)	(1, 1)	(20, 16, 500)	Same
BatchNorm (ReLU)				(20, 16, 500)	
Concat				(80, 16, 500)	
SpaConv5	20	(16, 1)	(1, 1)	(20, 1, 500)	0
BatchNorm				(20, 1, 500)	
Square				(20, 1, 500)	
AveragePool		(1, 75)	(1, 15)	(20, 1, 29)	0
Log				(20, 1, 29)	
Dropout				(20, 1, 29)	
Attention				(20, 1, 29)	
Flatten				580	
Classifier	580			2	

#### 2.3.1 Feature extraction layer

**Figure 2:**
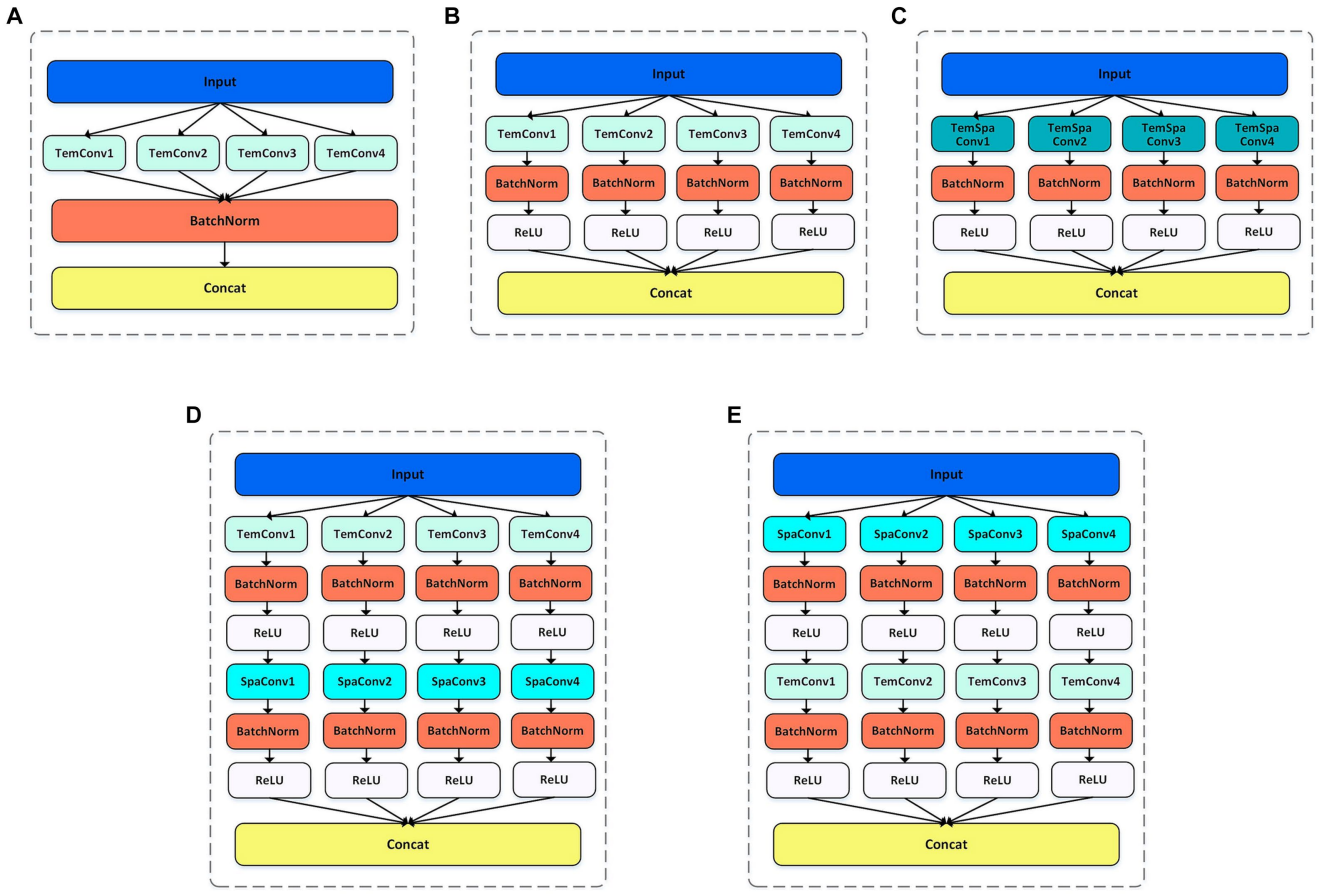
Five feature extraction structures. BR means adding BatchNorm and ReLU functions after the convolution. **(A)** TemConv: Temporal convolution. **(B)** TemConv+BR: temporal convolution followed by BR. **(C)** TemSpaConv+BR: temporal convolution and spatial convolution share a same convolution kernel and combined with BR. **(D)** TemConv+SpaConv+BR: temporal convolution followed by the spatial convolution and combined with BR. **(E)** SpaConv+TemConv+BR: spatial convolution followed by the temporal convolution and combined with BR.

Here, the multi-scale spatial and temporal feature extraction convolutions are combined to maximize the utilization of the spatiotemporal information in the EEG data. As shown in Figure 2, In order to obtain the best feature extraction layer structure, numerous ablation experiments, including five feature extraction modules within the multi-scale convolution structure, were designed to validate the efficacy of our proposed model for comparison. We conducted in-depth analysis on the spatiotemporal feature extraction module, and tried different combinations based on temporal convolution (44). In addition, batch normalization is introduced to enhance the consistency and stability of the model between different samples, and ReLU activation function is used to help the model better learn nonlinear features and improve the expression ability of the model. With these improvements, we expected to improve the performance and robustness of the model.

##### 2.3.1.1 Convolution + batch normalization + ReLU structure

Convolution + batch normalization + ReLU is a common feature extraction combination in deep learning, and has been successfully applied in some popular frameworks. The batch normalization layer speeds up the convergence of the network by normalizing each mini-batch. It reduces the internal covariance movement of each layer of input data and fixes its range to a smaller range, which helps the network learn effective feature representations faster. ReLU introduces a nonlinear activation function in the network, which does not cause gradient vanishing problems and can propagate gradients better than traditional activation functions such as sigmoid and tanh. The combined structure of Convolution + batch normalization + ReLU can accelerate convergence, improve generalization, mitigate gradient vanishing problems, and amplify the network’s expressiveness. Through the incorporation of batch normalization and ReLU modules after temporal convolution (Figure 2A), the model becomes more robust and has stronger feature extraction capabilities, as shown in Figure 2B.

##### 2.3.1.2 Spatial–temporal convolution

Temporal convolution can capture the temporal characteristics of the temporal evolution information, and the spatial convolution can capture the spatial characteristics between different channels. There are complex dynamic interactions between different brain regions in EEG signals, and spatiotemporal convolution can more effectively capture the dynamic connections and interactions between different channels in EEG signals than relying solely on temporal convolution. When the input is Channel × Time, a single convolution is employed to extract spatiotemporal features, only so that the kernel size is greater than 1 in both the temporal dimension and spatial dimension of the extracted features (i.e., C > 1 & T > 1, where C represents the kernel size of the spatial dimension and T represents the kernel size of the temporal dimension). Here, we referred to the Inception structure (multiple kernels of different sizes are used in the space–time dimension to capture features at different scales and levels of abstraction) as shown in Figure 2C. However, the results of spatiotemporal feature extraction using a single convolution prove to be suboptimal. In order to improve spatiotemporal feature extraction, we explored how to add spatiotemporal convolution to the model to obtain better results. Inspired by the idea of SqueezeNeXt model that decomposing 3 × 3 convolutional layers into 3 × 1 and 1 × 3 convolutional layers (45), the C × T of the original convolutional layer is decomposed into C × 1 and 1 × T. This decomposition scheme can not only reduce the number of parameters, increase the width and depth of the network, and capture long-range dependencies, but also increase the nonlinear feature extraction capability, thereby improving the efficiency and performance of the model.

By using two convolutions to extract spatial and temporal features, two different connection strategies were emerged. In the first way, the temporal features are extracted first, and then the spatial features are extracted, as shown in Figure 2D; In the second way, the spatial features are extracted first, followed by the temporal features, as shown in Figure 2E. Among them, in the first connection method, the temporal convolution section uses 10 filters with filter sizes of 64, 40, 26,16, and the spatial convolution part uses 20 filters with filter sizes of 8, 6, 4, 2, respectively. In the second connection method, 10 filters are used in the spatial convolution section and 20 filters are used in the temporal convolution section, and the filter size is consistent with the above.

In addition, the model also contains a layer of spatial feature convolution after the spatiotemporal feature convolution. This layer extracts spatial features while reducing the dimension of the feature map. Through such a design, we anticipated the model to comprehensively capture the spatiotemporal features in EEG signals, efficiently decrease computational complexity, and enhance the model’s overall performance and efficiency.

##### 2.3.1.3 Attention mechanism

Attention mechanism is a technology that emulates human attention processes, which has grown in significance within the domains of natural language processing and deep learning in recent years (46). The technology enables machines to handle large-scale data and complex tasks more intelligently by simulating human focus and the ability to selectively process information. At present, the attention mechanism has become a widely used tool for deep learning (47, 48). Integrating the attention mechanism module into the convolutional network can help it automatically select and focus on important features in the data, and improve the model’s ability to extract and represent key information. In this study, we employed three commonly used attention mechanisms: Squeeze-and-Excitation Networks (SE) (49), Convolutional Block Attention Module (CBAM) (50), and Efficient Channel Attention (ECA) (51). Among them, the relevant parameters of SE are set to: reduction = 1; the relevant parameters of CBAM are set to: reduction = 1, kernel_size = 7; and the relevant parameters of ECA are set to: kernel_size = 3. The principles of each of the three attention mechanisms are detailed below.

###### 2.3.1.3.1 SE

**Figure 3:**
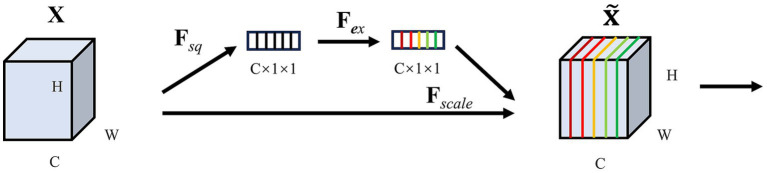
Structure of SE. $F_{\mathrm{scale}}$ represents channel-wise multiplication.

SE (Squeeze-and-Excitation Networks) is a convolutional neural network model designed to enhance the model’s ability to pay attention to crucial features from the input data. The core idea of SE is to add an attention module channel on top of the CNN. The module consists of two pivotal parts: a squeeze segment and an excitation segment, and its framework is shown in Figure 3.

Squeeze: SE uses global average pooling to compress each channel’s feature map into a scalar, which reduces the dimension of the feature map and captures global statistics between channels. If the input is a feature map $X\in R^{C\times H\times W}$ , the compressed feature map is $Z\in R^{C\times 1\times 1}$ , $Z_{C}$ is the c-th element of Z can be calculated as Equation (1):


(1) $$Z_{C}=F_{sq}\left(X_{C}\right)=\frac{1}{H\times W}\;\overset{H}{\underset{i=1}{\sum }}\overset{W}{\underset{j=1}{\sum }}X_{C}\left(i,j\right)$$

$F_{sq}$ represents the squeeze operation, where H and W denote the feature map’s height and width. In our EEG data, the channel and time correspond, respectively. $X_{C}\left(i,j\right)$ stands for the value on the feature map with a height dimension of i and a width dimension of j .

Excitation: to take advantage of the information gathered by squeeze, use excitation operations to capture channel dependencies. The excitation operation mainly obtains the attention weight S by nonlinear mapping by input of the compressed feature Z to the fully connected layer can be calculated as Equation (2):


(2) $$S=F_{\mathrm{ex}}\left(Z\right)=\sigma \left(W_{2}\delta \left(W_{1}Z\right)\right)$$

$F_{\mathrm{ex}}$ represents the excitation operation, δ represent to the ReLU function, $W_{1}\in R^{C/r\times C}$ and $W_{2}\in R^{C\times C/r}$ , r is the reduction radio. $W_{1}$ and $W_{2}$ are the weight parameters of the descending and ascending fully connected layer, and the σ represents the Sigmoid function, which limits the input value to the range of 0 and 1. The final output ${\tilde{X}}_{C}$ is derived from the feature map $X_{C}$ rescaling transformation as Equation (3):


(3) $${\tilde{X}}_{C}=F_{\mathrm{scale}}\left(X_{C},S_{C}\right)=S_{C}\cdot X_{C}$$

###### 2.3.1.3.2 CBAM

**Figure 4:**
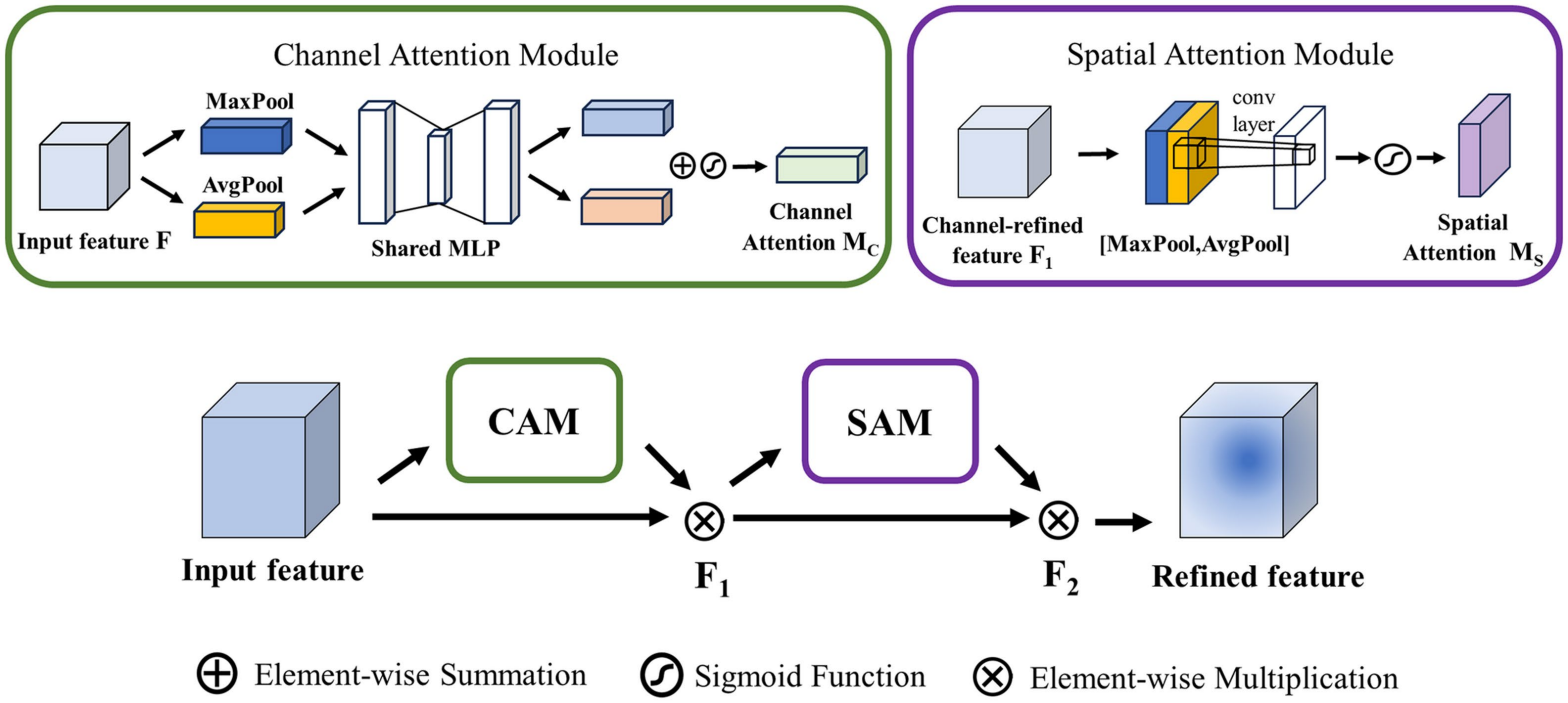
Structure of CBAM.

Convolutional Block Attention Module (CBAM) contains two submodules: the channel attention module (CAM) and the spatial attention module (SAM), as is depicted in Figure 4. CAM and SAM are used to strengthen the model’s attention capability to different channels and different spatial locations of the input feature map, respectively.

CAM: This module first obtains the average and maximum values of each channel by averaging pooling and maximizing pooling operations on the input feature map. These values are then processed by a hidden layer of Multilayer Perceptron (MLP) to learn and generate weights for each channel. Finally, the sum and merge of each element to obtain the channel attention degree $M_{C}\left(F\right)$ . For the input feature map $F\in R^{C\times H\times W}$ , after passing through the CAM $M_{C}\left(F\right)\in R^{C\times 1\times 1}$ can be calculated as Equation (4):


(4) $$M_{C}\left(F\right)=\sigma \left(MLP\left(\mathrm{AvgPool}\left(F\right)\right)+MLP\left(\mathrm{MaxPool}\left(F\right)\right)\right)$$

AvgPool signifies the average pooling operation, MaxPool signifies the maximum pooling operation, MLP stands for multilayer perceptron, and σ refers to the Sigmoid function.

SAM: This module is mainly concerned with the location of the information, which complements the CAM. To calculate spatial attention, the SAM uses average pooling and maximum pooling across the channel axis with convolution to generate spatial feature maps. Unlike channel attention, spatial attention does not use MLP , but instead employs convolution to process spatial feature maps. For input feature map $F_{1}\in R^{C\times H\times W}$ , after passing through the SAM $M_{S}\left(F_{1}\right)\in R^{1\times H\times W}$ can be calculated as Equation (5):


(5) $$M_{S}\left(F_{1}\right)=\sigma \left(f^{7\times 7}\left(\left[\mathrm{AvgPool}\left(F_{1}\right);\mathrm{MaxPool}\left(F_{1}\right)\right]\right)\right)$$

Where f stands for the convolution operation, 7 × 7 is the convolution kernel size, and σ refers to the Sigmoid function.

The final output feature map is calculated by CAM and SAM. The output map $M_{C}\left(F\right)$ after CAM is multiplied element by element with the input feature map F to generate feature $F_{1}$ , and $F_{1}$ is multiplied element by element with the output diagram $M_{S}\left(F_{1}\right)$ after SAM to generate the final output feature map $F_{2}$ .

###### 2.3.1.3.3 ECA

**Figure 5:**
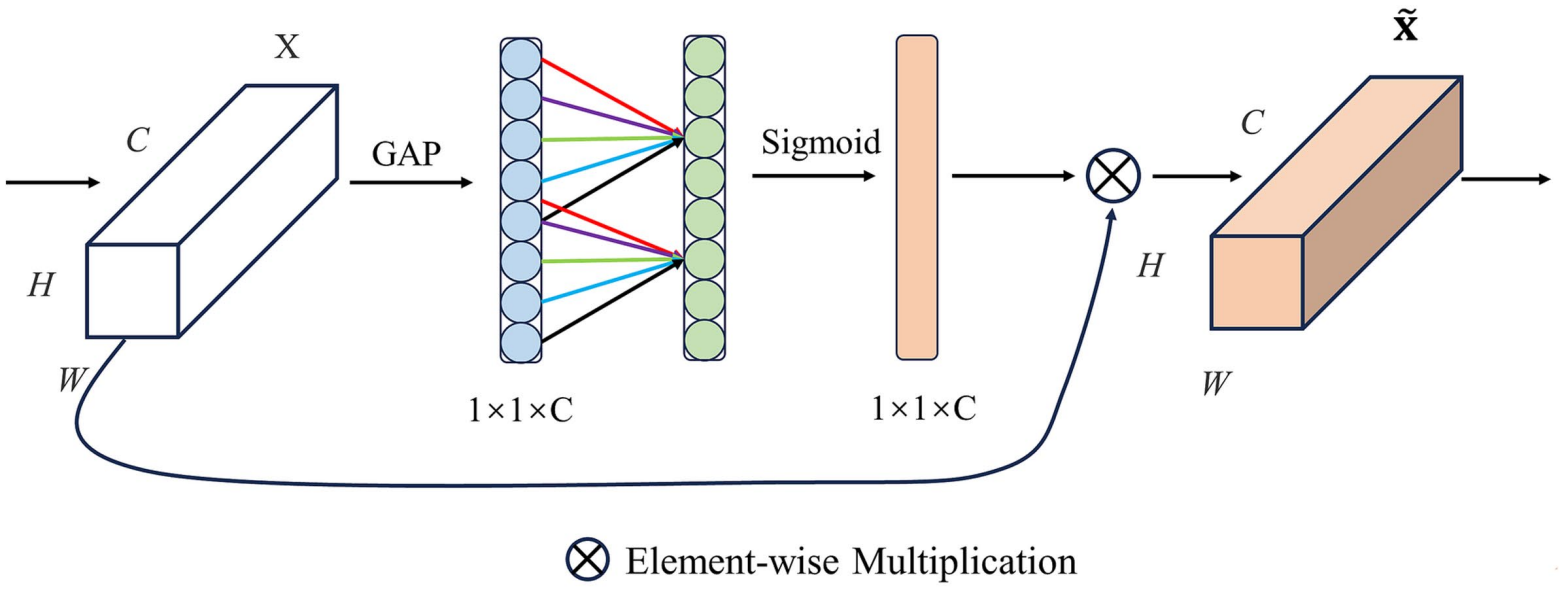
Structure of ECA.

Efficient Channel Attention (ECA) is commonly used in image classification tasks based on SE, as shown in Figure 5. The core idea of ECA is to use one-dimensional convolution operations to model relationships between channels instead of traditional fully connected layer operations, which can significantly reduce calculations, model parameters, and improve the calculation efficiency of the model. Similar to SE, ECA uses global average pooling (GAP) to aggregate spatial information for channels. Then, by performing a one-dimensional convolution operation on the feature map after global average pooling, all channels share learning parameters and quickly extract the relationship between channels, thereby enhancing the performance of channel attention which can be calculated as Equation (6):


(6) $$\omega =\sigma \left(C1D_{k}\left(\mathrm{GAP}\left(X\right)\right)\right)$$

C1D stands for one-dimensional convolution operation, k is the one-dimensional convolution kernel size, and σ is the Sigmoid function. The use of one-dimensional convolution not only reduces model complexity, but also ensures efficiency and effectiveness through local cross-channel interaction. Finally, ω is multiplied by X element by element results in the final feature map $\tilde{X}$ .

#### 2.3.2 Feature classification layer

The input of the feature classification layer is the feature map obtained after passing through the spatial feature convolutional layer. There are four steps in this layer. Firstly, the input feature map undergoes the application of the nonlinear function Square, and then downsampling is performed through the average pooling layer to reduce the dimensionality of the feature map while retaining the main feature information. Secondly, the nonlinear function Log for activation is used to extract features related to EEG bands after the averaging pooling layer. Thirdly, the dropout layer is introduced to prevent the model from overfitting. The dropout layer can randomly omit the output of some neurons during training, thereby reducing the dependence between neurons. Ultimately, the fully connected layer is utilized to finalize the classification.

### 2.4 Network training

**Figure 6:**
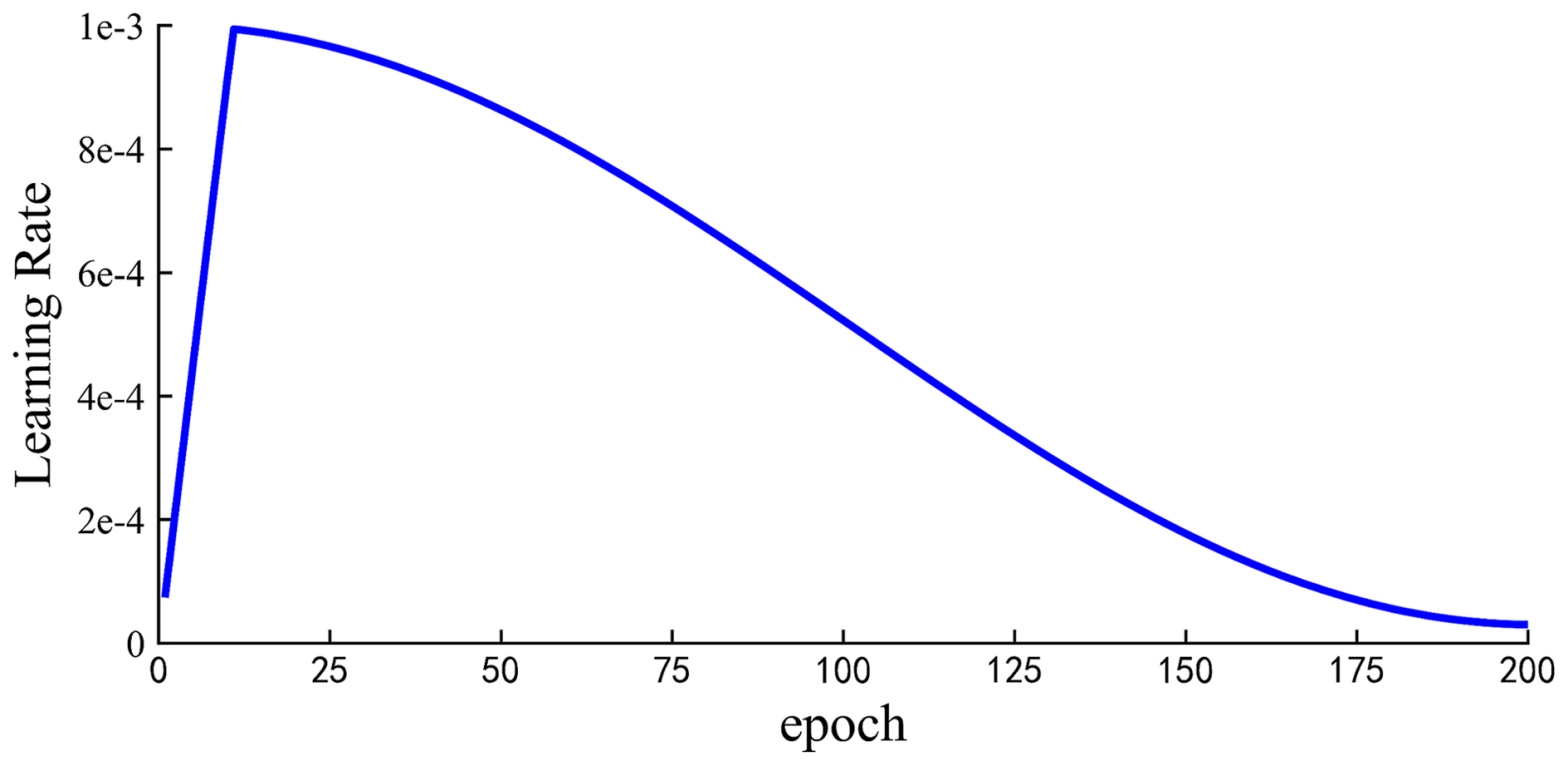
Learning rate setting during model training with warming strategy. Total trained in 200 epochs.

For the MSTCNN model, the batch size was set as 32 and the 200 epochs were trained for early stopping. Early stopping strategy was triggered when the value of the loss function no longer decreases in 10 consecutive epochs. CrossEntropy was chosen as the loss function, and AdamW optimizer was used for gradient optimization. In terms of the MSTCNN’s learning rate, the warm-up strategy was adopted shown in Figure 6, which starts with the learning rate set to 8e-5, gradually increases to 1e-3 after 10 warm-up rounds, and finally gradually decreases to 3e-5. By employing the learning rate warm-up strategy, the training speed can be accelerated, and the convergence and performance of the network can be improved. Applying a larger learning rate in the initial epochs can help the model find the global optimal solution or regions closer to the optimal solution in the parameter space more quickly. As the train continues execution, the learning rate gradually decreases, which is conducive to the training of stable networks.

### 2.5 Evaluation methods

Use cross-validation to evaluate the model’s performance and generalization ability. Nine folds of data were used for training and one fold of data for testing. Accuracy, Precision, Recall, and F_1_Score were computed to evaluate model performance as Equations (7–10). Specifically, True Positives (TP) indicates positive samples correctly classified, False Positives (FP) indicates negative samples incorrectly classified as positive, True Negatives (TN) indicates negative samples correctly classified, and False Negatives (FN) indicates positive samples incorrectly classified as negative.


(7) $$\mathrm{Accuracy}=\frac{TP+TN}{TP+TN+FP+FN}$$

(8) $$\mathrm{Precision}=\frac{TP}{TP+FP}$$

(9) $$\mathrm{Recall}=\frac{TP}{TP+FN}$$

(10) $$F_{1}\;\mathrm{Score}=\frac{2TP}{2TP+FP+FN}$$

## 3 Results

The results of different multi-scale convolutional structures for GAD detection are given in Table 1. The model with only temporal convolution obtained an accuracy of 96.75%, a precision of 96.69%, a recall of 97.68% and a F_1_Score of 97.18%. In order to enhance the generalization ability and nonlinear expression ability of the multi-scale CNN model in convolutional feature extraction, Convolution + batch normalization + ReLU structure was added in the model. The accuracy improved to 98.25%. Therefore, all other comparison models adopted the Convolution + batch normalization + ReLU structure. Further, we introduced spatial convolution and explored different combinations of temporal and spatial convolution for comparisons. The results showed that the combination with spatial + temporal convolutions (named MSTCNN) yielded superior performance, achieving an accuracy of 99.19%, a precision of 99.45%, a recall of 99.14% and a F_1_Score of 99.29%.

Several classic models also used to verify the effectiveness of our model. The compared models of EEGNet, multi-resolution CNN (MRCNN), and CNN-LSTM, yielded average accuracies of 94.34 ± 0.75%, 96.35 ± 0.42%, and 97.26 ± 0.86% on our datasets, respectively. The specific classification evaluation indicators of each model are shown in Table 2.

**Table 2:** Classification performances of different convolution methods.

Models	Accuracy (%)	Precision (%)	Recall (%)	F_1_ Score (%)
TemConv	96.75 ± 0.68	96.69 ± 1.20	97.68 ± 0.65	97.18 ± 0.56
TemConv + BR	98.25 ± 0.35	98.19 ± 0.51	98.76 ± 0.47	98.47 ± 0.31
TemSpaConv + BR	97.43 ± 0.85	98.19 ± 0.99	97.33 ± 1.56	97.75 ± 0.73
TemConv + SpaConv + BR	98.64 ± 0.32	98.75 ± 0.71	98.88 ± 0.52	98.81 ± 0.26
SpaConv + TemConv + BR (MSTCNN)	**99.19 ± 0.40**	**99.45 ± 0.47**	**99.14 ± 0.49**	**99.29 ± 0.34**

“TemConv” means temporal convolution. “BR” means adding BatchNorm and ReLU functions after the convolution. “TemSpaConv” means that the temporal convolution and the spatial convolution are in the same convolutional kernel. “TemConv + SpaConv” means the temporal convolution followed by the spatial convolution. “SpaConv + TemConv” means the spatial convolution followed by the temporal convolution. The bold values provided in the table represent the best results compared with others.

Based on our proposed convolutional structure (SpaConv + TemConv + BR), three commonly used attention mechanisms (SE, CBAM, and ECA) were added into the model. As shown in Table 3, our MSTCNN model shows performance improvement following the inclusion of attention mechanisms and yielded more stable results. In particular, the improvement effect of the SE attention mechanism was the most significant, with the highest accuracy of 99.48%.

**Table 3:** Classification performances of classical deep learning models.

Models	Accuracy (%)	Precision (%)	Recall (%)	F_1_ Score (%)
EEGNet (52)	94.34 ± 0.75	95.80 ± 1.23	94.26 ± 2.02	95.00 ± 0.71
MRCNN (53)	96.35 ± 0.42	96.28 ± 1.22	97.40 ± 1.46	96.82 ± 0.44
CNN-LSTM (54)	97.26 ± 0.86	98.32 ± 1.01	96.89 ± 2.14	97.57 ± 0.81
Our model	**99.19 ± 0.40**	**99.45 ± 0.47**	**99.14 ± 0.49**	**99.29 ± 0.34**

The bold values provided in the table represent the best results compared with others.

Besides, the impacts of five different frequency bands (Theta, Alpha1, Alpha2, Beta1, and Beta2) were explored on the classification of GAD and HC with MSTCNN-SE model. As indicated in Table 4, the accuracy of the Theta band and the Alpha1 band is lower with a classification accuracy of less than 90%. With the increase of frequency band, the classification accuracy also gradually improved, and the highest classification accuracy of 97.45% was achieved on the Beta2 band.

**Table 4:** Classification performances of different attention mechanisms.

Models	Accuracy (%)	Precision (%)	Recall (%)	F_1_ Score (%)
MSTCNN-SE	**99.48 ± 0.23**	**99.66 ± 0.23**	99.43 ± 0.28	**99.55 ± 0.20**
MSTCNN-CBAM	99.34 ± 0.38	99.31 ± 0.54	**99.54 ± 0.33**	99.42 ± 0.33
MSTCNN-ECA	99.46 ± 0.20	99.61 ± 0.22	99.44 ± 0.46	99.52 ± 0.18

MSTCNN-SE/CBAM/ECA means adding SE Attention, CBAM Attention, or ECA Attention for MSTCNN model. The bold values provided in the table represent the best results compared with others.

Based on the results of Table 4, that is, high accuracy can be obtained with the high-frequency EEG rhythm. Three high-frequency EEG bands, including 13-30 Hz, 10-30 Hz, and 8-30 Hz, were extracted for GAD diagnosis. The results are presented in Table 5. It shows that 10-30 Hz can gain consistent accuracy compared with 4-30 Hz, which has no statistically significant difference (see Table 6).

**Table 5:** Classification performances of different frequency bands with MSTCNN-SE model.

Frequency band	Accuracy (%)	Precision (%)	Recall (%)	F_1_ Score (%)
Theta	88.09 ± 1.09	89.08 ± 2.30	90.34 ± 2.16	89.66 ± 0.99
Alpha1	86.35 ± 1.12	88.53 ± 1.93	87.52 ± 2.17	87.99 ± 1.10
Alpha2	93.56 ± 0.76	93.45 ± 1.05	95.46 ± 1.54	94.43 ± 0.67
Beta1	96.26 ± 0.48	96.69 ± 1.08	96.79 ± 0.72	96.73 ± 0.41
Beta2	97.45 ± 0.43	98.08 ± 0.86	97.46 ± 1.19	97.76 ± 0.41

**Table 6:** Classification performances of extended frequency bands with MSTCNN-SE model.

Frequency band	Accuracy (%)	Precision (%)	Recall (%)	F_1_ Score (%)
13–30 Hz	98.90 ± 0.29	99.13 ± 0.34	98.95 ± 0.56	99.04 ± 0.25
10–30 Hz	**99.47 ± 0.24**	**99.48 ± 0.37**	**99.59 ± 0.28**	**99.54 ± 0.20**
8–30 Hz	99.42 ± 0.26	**99.48 ± 0.47**	99.52 ± 0.29	99.50 ± 0.22

The bold values provided in the table represent the best results compared with others.

## 4 Discussion

This study proposed a novel end-to-end multi-scale Spatial–Temporal local sequential and global parallel convolutional neural network called MSTCNN and applied it to diagnose GAD by utilizing multichannel EEG signals. The main findings are as follows. Firstly, the proposed MSTCNN combined with SE attention mechanism obtained an excellent classification performance on the collected EEG data, with an accuracy of 99.48%, a precision of 99.66%, a recall rate of 99.43%, and a F1 Score of 99.55%. Secondly, an interesting phenomenon was stumbled upon: the high-frequency band holds significant importance in diagnosing GAD, and higher frequency band can obtain higher accuracy in GAD recognition. Notably, the accuracy of the 10-30 Hz band is consistent with the 4-30 Hz band. Detailed discussion will be presented next.

### 4.1 Best classification performance from MSTCNN model

When applying deep learning to extract features from EEG signals, researchers mostly focus on multi-scale convolution in the temporal domain and ignore the spatial relationships between channels (42–44). Introducing multi-scale spatial convolution can extract spatial features more efficiently, thereby improving model performance. In this study, we explored the method of multi-scale spatial–temporal convolution and found that the spatial axis decomposition idea of splitting a single convolution kernel into two convolutions can achieve better results. This idea can not only effectively reduce the complexity of the model and decrease the risk of overfitting, but also improve the computational efficiency (45). Furthermore, we compared the effects of convolutional sequences with different spatial and temporal convolutions. It has been presented in Table 1 that the accuracy of spatial convolution combined with temporal convolution is 0.55% higher than that of temporal convolution combined with spatial convolution. Since there is spatial convolution after the spatial–temporal convolution module, it can effectively avoid redundant operations in the spatial dimension.

We also tried to validate the effectiveness and accuracy of our proposed MSTCNN Model for GAD detection. On the one hand, some classical deep learning models was used to compare with our models. Among them, EEGNet is a concise deep learning model commonly used to process EEG data, which can efficiently extract features and use them for classification (52). In our study, EEGNet model obtained an accuracy of 94.34%. Next, we tried the MRCNN model proposed by Eldel et al. for sleep EEG data (53), and its accuracy in our classification task reached 96.35%. Finally, CNN-LSTM model proposed by Wang et al. (54) was used to classify our data, and obtained an accuracy of 97.26%. The above results indicate that the multi-scale spatial–temporal convolution strategy proposed in this study outperforms conventional deep learning models, leading to exceptional achievements. On the other hand, our results were compared with other similar studies. Park et al. used machine learning in major psychiatric disorders based on resting EEG and obtained an accuracy of 91.03% (55). Al-Ezzi et al. used a deep learning model (CNN-LSTM) for three different degrees of anxiety and HC based on task-state EEG data, and obtained the accuracy of 92.86%, 92.86%, 96.43%, and 89.29%, respectively (37). Mohan et al. used CNN to discriminate depressed and anxiety patients based on EEG and obtained an accuracy of 97.6% (56). It is worth mentioning that our previous study, combining features extraction and machine learning model, obtained an accuracy of 97.83% for GAD and HC (20). MSTCNN model, to the best of our knowledge, has achieved the highest accuracy for GAD and HC detection compared with advanced models and existed studies. In summary, MSTCNN has outstanding advantages in classification performance. These findings not only verify the effectiveness of our proposed model, but also provide support for its potential advantages in subsequent clinical application for GAD diagnosis.

### 4.2 MSTCNN improved with attention mechanisms

EEG signals contain a wealth of information, which poses challenges to signal processing, feature extraction, and classification. To efficiently extract features and obtain excellent classification performance, the attention mechanisms were employed in combination with MSTCNN. Specifically, we incorporated and evaluated three widely used attention mechanisms (SE, CBAM, and ECA) into the convolution. At present, the attention mechanism has gradually become a boom in deep learning, and an increasing number of researchers are applying it to EEG signal processing. Deng et al. (57) improved the accuracy of major depressive disorder classification from 91.24% to 94.37% by adding SE attention mechanism to one-dimensional convolution. Chen et al. used CBAM attention for ResNet34 in emotion recognition task, and the accuracy increased by 5.54% compared with ResNet34 (58). Jia et al. (59) proposed a spectral-temporal convolutional neural network with ECA attention, and the classification results showed that there was also a significant increase for the classification performance. By introducing these attention mechanisms, MSTCNN model can focus on more important features, further optimize the feature extraction process and enhance the performance and stability of the model.

### 4.3 Deep learning reveal the key frequency band for GAD diagnosis

Previous studies have reported a clear correlation between EEG rhythms and alternate EEG features in GAD patients (60). Additionally, our previous research has pointed to the importance of beta rhythms in GAD (20). Significantly higher accuracy was obtained for Beta rhythms in the high-frequency band compared to Theta and Alpha in the low-frequency band. Beta rhythms are associated with functions such as attention, cognitive control, and emotion regulation in the brain (61). Given that GAD often accompanies mood fluctuations, which may be the reason why beta sub-bands are prone to exhibit high accuracy in GAD and HC classification. In summary, different frequency bands had a significant impact on the classification results of GAD. A more universal regularity is that the higher the frequency range, the better the GAD classification performance.

Based on the above findings, we attempted to expand the frequency bands to further explore key frequency bands for distinguishing GAD. Three extended frequency bands are extracted in this study: 13–30 Hz, 10–30 Hz, and 8–30 Hz. In contrast to the results of Beta2, the classification accuracy is considerably improved when using the 10-30 Hz frequency band with the accuracy of 99.47%, which has no statistical difference with the accuracy of the 4-30 Hz frequency band (*F* = 0.0099, *p* = 0.92; which was tested by one-way analysis of variance. If *p* is less than 0.05, there is a significant difference between groups. Otherwise, there is no significant difference). Wen et al. used the CNN model and EEG signals to identify cognitive impairment diseases, and also achieved the highest classification accuracy through three frequency band combinations (10–30 Hz) compared with other combinations (62). To the best of our knowledge, no prior research has utilized deep learning methods to explore the impact of different combined frequency bands on GAD classification. Our current results provide preliminary evidence supporting the importance of high-frequency bands in GAD identification and highlight the prominent advantages of the 10-30 Hz band. These findings will contribute to a more comprehensive understanding of the relationship between EEG frequency bands and GAD, and provide a new insight for the GAD diagnosis. The excellent classification performances of GAD detection at high frequencies can provide guidance for subsequent practical applications. For instance, we may choose to filter out low frequencies to effectively mitigate the noise and interference stemming from those bands when developing an EEG-based system for GAD diagnosis.

### 4.4 Limitation

Although the MSTCNN proposed in this study has demonstrated impressive capabilities in the identification of GAD and HC, it still has come with certain limitations. Firstly, the main manifestation is the sample size utilized in the study is relatively limited (45 participants for GAD and 36 participants for HC), which limits our effective verification of the robustness and generalization ability of the model. Secondly, our deep learning model appears to lack reasonable interpretability for GAD diagnosis. Thirdly, in real-life scenarios, the process in which hospitals collect EEG data may be some discrepancies, such as different EEG acquisition equipment and inaccurate placement of electrodes, which may lead to diagnostic performance decline. To more comprehensively evaluate the performance and generalization ability of the model, we will try to use more diverse data sources and explore deep learning model interpretability in follow-up studies.

## 5 Conclusion

In this study, an end-to-end deep learning MSTCNN model was proposed for the precise diagnosis of GAD based on EEG signals. Three widely used attention mechanisms were applied on MSTCNN model for the improvements of the classification performances. And different frequency bands were extracted to explore key frequency band in GAD diagnosis. Notably, MSTCNN combined with the attention mechanism of Squeeze-and-Excitation Networks achieved an excellent classification performance, to the best of our knowledge, with the highest accuracy of 99.48%. More interestingly, it is found that higher frequency band can obtain higher accuracy in GAD recognition. The accuracy of the high-frequency band with 10-30 Hz has no statistical difference with the accuracy of the 4-30 Hz frequency band. This finding could simplify the signal processing process and reduce the complexity of low-frequency EEG data processing. In sum, this work can have a positive impact on the precise diagnosis of GAD and move a step forward towards the automatic diagnostic system of GAD.

## Data availability statement

The raw data supporting the conclusions of this article will be made available by the authors, without undue reservation.

## Ethics statement

The studies involving humans were approved by Ethics Committee of Zhejiang Normal University. The studies were conducted in accordance with the local legislation and institutional requirements. The participants provided their written informed consent to participate in this study.

## Author contributions

WL: Writing – original draft. GL: Writing – review & editing. ZH: Writing – original draft. WJ: Writing – review & editing. XL: Writing – review & editing. XX: Writing – review & editing.
